# Methodology of DNA extraction and sequencing from living cardiomyocytes collected by catheter in humans

**DOI:** 10.1016/j.gimo.2025.103473

**Published:** 2025-11-18

**Authors:** Flavie Ader, Céline Guilbeau-Frugier, Emeline Lhuillier, Frédéric Martins, Anne Alicia Gonzalez, Anne Rollin, Maxime Beneyto, Céline Gales, Filipe Pires, Jean-José Maoret, Jean-Michel Sénard, Jean Timnou-Bekouti, Eric Villard, Laetitia Duboscq-Bidot, Estelle Gandjbakhch, Philippe Maury

**Affiliations:** 1 INSERM, UMR_S 1166, Sorbonne Université, Paris, France; 2 APHP- Sorbonne Université, Unité Fonctionnelle de Cardiogénétique et Myogénétique Moléculaire, Service de Biochimie Métabolique, CHU Pitié Salpêtrière- Charles Foix, Paris, France; 3 Université Paris Cité UFR de Pharmacie, Département 3, Université de Paris, Paris, France; 4 Department of Cardiology, University Hospital Rangueil, Toulouse, France; 5 I2MC, INSERM UMR 1297, Toulouse, France; 6 GeT-Santé, Plateforme Génome et Transcriptome, GenoToul, Toulouse, France

**Keywords:** Arrhythmogenic right ventricular cardiomyopathy, Cardiomyocyte, Epigenetics, Mosaicism, Single-cell genetical analysis

## Abstract

**Purpose:**

We present here the technical feasibility of percutaneously retrieving cardiomyocytes (CMs) through the lumen of irrigated ablation catheters, with the aim of obtaining DNA of sufficient quality/quantity for allowing DNA amplification, screening, and derived genetic analysis.

**Methods:**

Irrigated conventional catheters for ablation were used for creating endocardial right ventricular voltage maps in 38 patients with suspected or proved arrhythmogenic right ventricular cardiomyopathy. Blood material was collected from scar areas by aspiration and filtered, CMs detected by light microscopy were aspirated, centrifugated, and freezed.

DNA was extracted, amplified, and sequenced, and variants were compared with variants obtained from leukocyte DNA.

**Results:**

At least 1 CM was obtained in 95% of patients (median 11 CM/patient). After refinements of the technique, a total of 136 samples (22 patients) allowed DNA extraction and amplification, successful in 60% of samples (16 patients). DNA capture sequencing of a panel of cardiomyopathy-associated genes was successfully performed in 14 patients and compared with blood sequencing in 11. After controlled by Sanger, an additional variant, not present in blood, has been confirmed in CM in one patient.

**Conclusion:**

This new mini-invasive technique of sampling allows to perform genetic analysis on CMs. Pending future improvements, this technique could provide new sources of human cells for research and potential mosaicism detection.

## Introduction

Obtention of living cardiomyocytes (CMs) is the mandatory condition for any genomic, transcriptomic or proteomic study on cardiac cells. Noncardiac cellular lineages may be used for DNA analysis, such as lymphocytes, oral mucosa, or cutaneous cells.^1^ However, more advanced genomic studies on mosaicisms^2^^,^^3^ or epigenetics^4^ involving tissue-specific DNA modifications need acquisition of living organ-derived cells.

Because cardiac cells are not present in the circulating blood pool, CMs could only be acquired by percutaneous or surgical cardiac biopsy. Surgical obtention of CMs cannot be performed outside therapeutical procedures for evident reasons. Even if relatively easily performed in clinical practice, percutaneous cardiac biopsy is an invasive procedure that may entail significant morbidity and mortality, carrying the risk of arrhythmias, conduction disturbances or cardiac perforation of several percents.^5^ Moreover, standard percutaneous cardiac biopsies are usually performed at fixed locations (ie, the right interventricular septum, which may be remote from diseased areas), whereas biopsies at more specific areas of interest using steerable sheaths are restricted to experienced operators/centers with increased risks for cardiac damages.^6^

Another potential solution for obtaining living CMs would use the lumen of irrigated steerable catheters utilized for percutaneous radio-frequency (RF) ablation of cardiac arrhythmias.^7^ Externally irrigated tip catheters are the standard of care in percutaneous RF ablation because allowing cooling of the catheter tip for delivering enough RF power. Because the inner lumen of irrigated tip catheters may reach 500 μm in diameter, we assumed that cells such as CMs may pass through and could be collected through direct aspiration.

The technical feasibility of percutaneously retrieving CMs through the lumen of irrigated ablation catheters, with the aim of obtaining DNA of sufficient quality and quantity for subsequent DNA sequencing has been recently demonstrated by our group.^8^ This article is now developing and detailing our learning curve and the methodology used.

## Materials and Methods

A total of 38 patients referred at our institutions for invasive investigation or percutaneous ablation of suspected or previously diagnosed arrhythmogenic right ventricular cardiomyopathy (ARVC) were prospectively included (27 males, 53 ± 14 yo).

As part of diagnosis or therapeutic purposes in our institutions,^9^ endocardial right ventricular voltage maps were created using intracardiac catheters. Briefly, a 3D mapping system was used to build anatomic maps of the right ventricular cavity by collecting endocardial points recorded by the roving ablation catheter, while adding some information about voltage at each point. Diseased areas or scars (bipolar voltage < 1.5 mV, with or without fragmented or delayed local electrical potentials) were delineated on the voltage map, which may help in diagnosis of ARVC.^9^ For the ARVC patients referred for ablation of ventricular arrhythmias, similar endocardial voltage maps were performed before ablation for locating and ablating areas involved in arrhythmias.

Externally irrigated conventional (nonsurrounding flow) Smartouch or Thermocool catheters (Biosense Webster Inc) were mainly used for attempting to retrieve cells because other catheters available on the French market presented with narrower inner lumen or irrigation holes, failed, or were not tested.

All patients received intravenous heparin (dosing according to the type of procedure). Baseline irrigation with 2 cc/mn rate (normal 0.9% saline) was used for avoiding the catheter to be occluded by thrombus. Low voltage areas were targeted for retrieving cardiac cells in each patient. The irrigated catheter was firmly positioned on the endocardial scar surface to obtain good contact with tissue. Then, manual aspiration with a 10-ml syringe was performed directly at the proximal end of the catheter through the saline input port. First cc were eliminated. A second aspiration was performed to collect between 2 and 4 ml of blood material. This material was then flushed into a 70-μm pore filter, as well as the material still present inside the lumen of the catheter, once removed and flushed with saline. All of the operation takes a few minutes and can be performed again at the same spot or at another location.

Filter was then immersed 3 times into a 37 °C standard cell culture medium (Gibco by Life Technologies, MEM(1X)+GlutaMAX) added with blebbistatin (25 μM) for eliminating cell contractions and delaying cellular death, before being rinsed with the same medium to minimize contamination by blood cells. Finally, the filter was turned back over a Petri dish and rinsed again for flushing the cellular material into the dish, before being immediately transported at 37 °C temperature into the laboratory for light microscope analysis (×10) and cell counting.

At this stage, we evaluated number and quality of CMs. All visualized CMs were then manually aspirated with a micropipette, then the material was centrifugated (1 min, 0.2 rcf), the supernatant was removed, and the pellet containing CMs was freezed at −80 °C. After the 14 first patients, the pellet was sometimes transferred into several tubes (defined below as “samples”) because of too much remaining supernatant, hindering the extraction/amplification processes.

**Figure 1:**
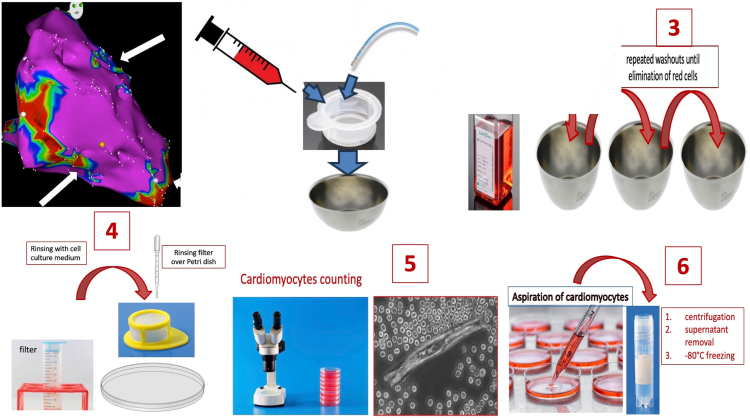
**Schematic representation of the sampling process**.

A schematic representation of the whole sampling process is depicted in Figure 1. This procedure was duplicated for any additional sample collection from different areas of interest. To demonstrate that CMs came from the targeted areas and were not present in the circulating blood, control samples were also collected, consisting of aspirated blood from inside the right atrium by the same catheter.

Additionally, conventional DNA analysis was also performed on blood leukocytes to validate the concordance between blood and CMs sample, and to identify potential additional variants in CMs DNA.

### DNA generation

DNA issued from CMs were extracted and amplified using the GenomePlex Single-Cell Whole Genome Amplification Kit according to manufacturer use (Sigma-Aldrich, WGA4-10RXN). Pellets were suspended in Tris-EDTA buffer for a final volume of 9 μl.

Briefly, we first lysed the nucleus and removed the proteins by incubating cells at 50 °C for 1 hour on mixture Proteinase K-Single-Cell Lysis and Fragmentation Solution. The genomic DNA was fragmented for 4 minutes at 99 °C. A set of random primers linked with common adaptors was annealed to the fragmented DNA template at the following series of temperatures: 16 °C for 20 minutes, 24 °C for 20 minutes, 37 °C for 20 minutes, 75 °C for 5 minutes, and 4 °C hold. Then, polymerase chain reaction was performed to amplify the fragmented DNA with an initial denaturation at 95 °C for 3 minutes, and 25 cycles of 94 °C for 30 seconds and 65 °C for 5 minutes. The concentration was determined by fluorescence (Clario Star) and the product size using a High Sensitivity NGS kit (DNF-474, Agilent) on Fragment Analyzer or D1000 on Tape Station (Agilent).

### DNA sequencing

DNA could only be sequenced if the concentration was >1 ng/uL of double-strand DNA assessed by fluorescence (Clariostar, Labtech). Extraction failure was considered for DNA concentrations < 1 ng/uL. DNA generated was sequenced using a targeted custom panel (100 kb) including all coding and flanking intronic regions (±10 bp) of 55 genes responsible of ARVC and inherited cardiomyopathies and cardiac arrhythmias (list of genes available in Supplemental Table 1).

For DNA extracted from CMs, custom targeted gene enrichment and DNA library preparation were performed using the NimbleGen EZ choice probes and Kappa Library preparation adapted (Nimblegen, Roche Diagnostics). Taking into consideration the low concentration of extracted DNA, we used the shorter fragmentation time recommended (20 minutes) and the maximum number of cycles recommended for Pre-LM polymerase chain reaction step (8 cycles).

For DNA extracted from leukocytes (Qiagen), the same reagents have been used for library preparation according to manufacturer recommendations.

DNA libraries were pooled by 8 and sequenced by 24 using the Illumina MiSeq platform on a 500 cycle Flow Cell (Illumina) and MiSeq Software generates FASTQ format files after demultiplexing patients’ sequences. Merged single reads and paired-end reads were then aligned on GRCh37 (hg19) human reference genome, using BWA-MEM. Variant calling was performed using the pipeline previously described.^10^

### Validation of variants

All genetic variants with a mean coverage of 30x and a variant allele frequency of 30% were considered. Variants sequenced from CM-DNA were compared with variants obtained from leukocyte DNA. Comparison allowed to validate the concordance between blood and CMs samples and to identify potential additional variants in CMs DNA. We focused on coding region and splicing regions (exon ± 10 bp) to avoid noise in repeated regions.

If additional variant has been identified in CMs, Sanger sequencing was performed using custom primers to amplify and sequence short fragments (maximum 200 bp) as DNA from cardiocytes present a size around 400 bp because of the extraction process. Detection of copy-number variants has not been performed for CMs.

Informed consent was obtained from all patients. This study was approved by a national ethical committee (Comité de Protection des Personnes du Sud Ouest @ Outre Mer IV) (n° CPP16-026a) and registered at our institution under the n° 15 7731 02.

## Results

Some of the results presented here had been already published^8^ but without procedural details.

### Sample collection

Samples were performed from 1 to 2 targeted right ventricular scar areas for each patient. From this sampling procedure, several tubes named in following text “samples” have been prepared.

**Figure 2:**
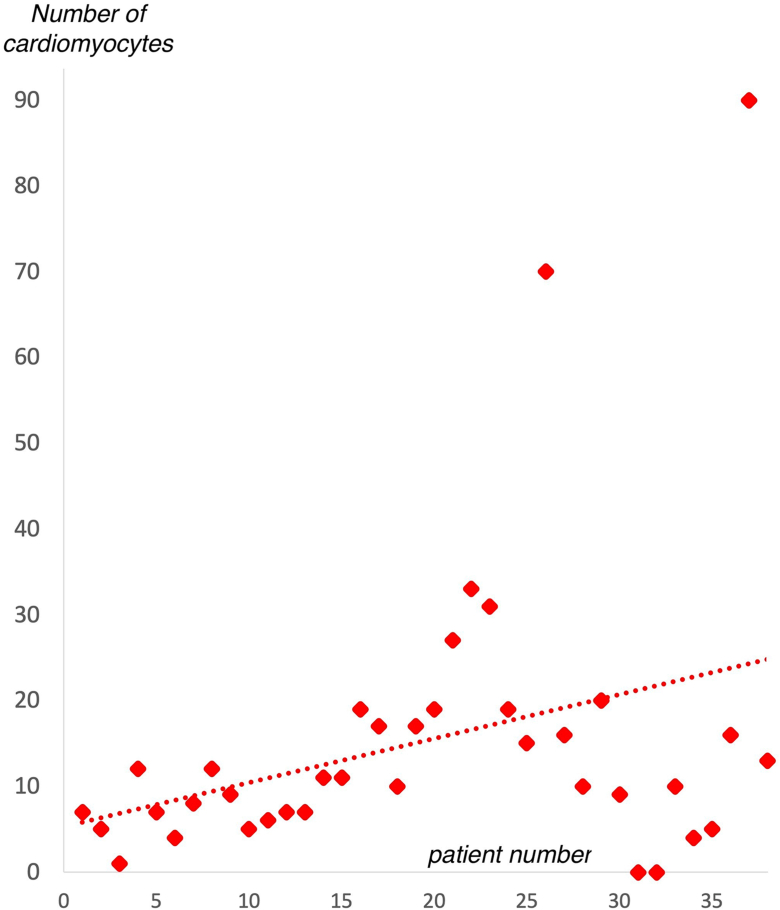
**Improvement in the number of collected cardiomyocytes with the learning curve**.

At least 1 CM was obtained in 36 out of the 38 patients (success rate 95%) with a median of 11 CMs per patient (1 to 90). No complication occurred due to the sampling process. The number of CMs increased with the experience of the operators (Figure 2). No CM was detected in control samples from right atrium in any patient.^8^

**Figure 3:**
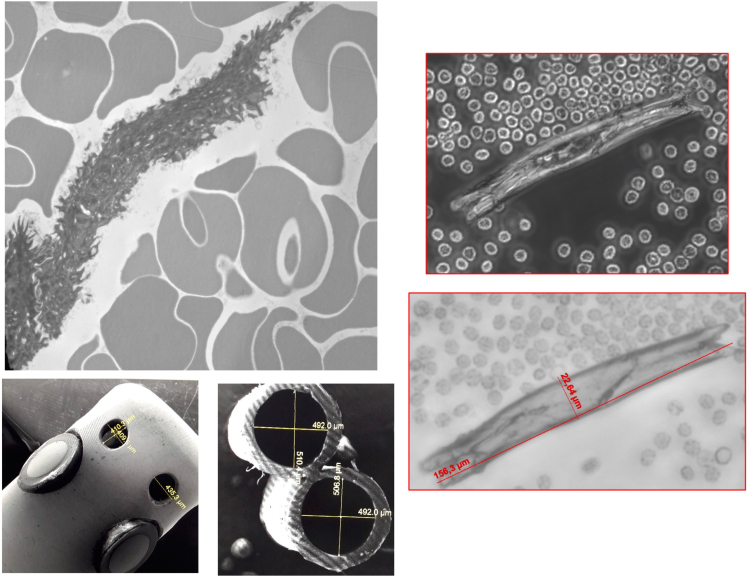
**Microscopic view of retrieved cardiomyocytes compared with the size of catheter lumen and pores**.

Microscopic view of retrieved CMs can be seen in Figure 3, as well as CM size compared with the lumen and pores of the catheter used.

### Sample preparation

A flowchart of the complete collection of samples, extraction, and sequencing can be found in the princeps publication.^8^ Details about patients and samples can be found in Supplemental Table 2.

In the first 14 out of the 36 patients with retrieved CMs, too much supernatant remained before DNA extraction, avoiding effective single-cell DNA whole genome amplification.^8^ After refinements of the isolation technique, a total of 136 samples containing CMs from 22 patients have been collected (median 5 samples per patient, 3 to 29).^8^

For 6 out of the 22 patients, DNA extraction failed, and none of the 62 samples did reach the quantity required for sequencing. Thus, extraction and amplification led to 82 out of the 136 samples (60%) qualified for sequencing (DNA concentration > 1 ng/uL). The DNA quantity ranked from 30 ng to 3810 ng/sample qualified for sequencing.

The 74 remaining samples from 16 patients have been treated in 2 sets: a first extraction and sequencing RUN 1 (in 2020) and a second set of samples (extraction and sequencing RUNS 2 and 3 in 2023), corresponding to a mean of 3390 ± 420 ng of DNA per sample from RUN 1 and 60.8 ± 39 ng for RUNS 2 and 3. Quality of sequencing runs is shown in Supplemental Table 3.

### CM-sample sequencing

DNA capture sequencing of the panel of cardiomyopathy-associated genes was successfully performed for 14 of 16 patients after single-cell DNA extraction and whole genome amplification, with a total of 52 samples sequenced for the 3 runs (16 for RUN 1 and 36 for RUNS 2-3). For 2 patients, library preparation before capture and sequencing failed.

Comparison of variants from leukocytes and CMs could be performed for 11 of these 14 patients (blood genetical screening judged finally clinically inappropriated in 3) (in total, 43 samples with a median 5 samples per patient [1 to 9]).

**Figure 4:**
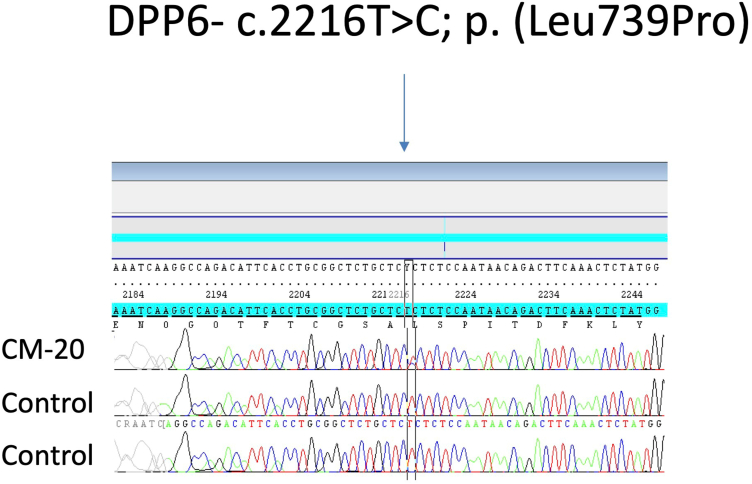
**Sanger sequencing electrophoregram for patient 20 blood (control) sample and cardiomyocyte sample (CM20).***DPP6* (HGNC:3010): NC_000007.13:g.154677425T>C, NM_130797.4: c.2216T>C p.(Leu739Pro) is detected on cardiomyocytes but not in the blood.

We first confirmed that samples of CMs and leukocytes were paired in each patient. For variants identified only in cardiomyocytes, the bam files alignment in IGV allowed us to remove the potentials artefact. Afterward, we tested by Sanger sequencing a total of 14 variants identified in CM samples but not in blood samples (see Supplemental Table 4).^8^ We confirmed the presence of an additional variant in CMs in comparison with blood in 1 case^8^ (see Sanger sequencing electrophoregram in Figure 4 and variant allele frequency in Supplemental Table 5). We also collected oral mucosa cells from this patient, but Sanger sequencing failed on the extracted DNA from this sample.

## Discussion

We describe here in more details a novel mini-invasive technique to obtain in vivo human living adult CMs.^8^ We demonstrated that the technique was feasible and reproducible in routine practice after a short learning curve. Furthermore, we showed that DNA of sufficient quality for allowing genetic analysis may be extracted from these CMs.

As an opportunity to obtain CMs, this technique may represent an important step forward in translational research of heart diseases. One major limitation in clinical research and practice remains the access to heart tissue/cells to study human cardiac diseases, particularly in the setting of inherited cardiomyopathies. From several years now, the development of induced pluripotent stem cells-derived CMs has allowed to have access to a human in vitro model of cardiomyopathies. However, this model still suffers from several limitations: lack of maturation, absence of the endocrine environment, and dependence on culture conditions. The possibility to have directly access to the patient CMs using a mini-invasive technique may have a large number of direct applications, especially with the development of single-cell technology, such as single-cell transcriptional, epigenetic, or proteomics analysis.

According to out technique, at least 1 CM could be obtained in 95% of patients, with a median of 11 CMs per patient, increasing with the experience of the operators. Then, DNA extraction and amplification led to 60% of samples qualified for sequencing. Finally, DNA sequencing could be successfully performed for 87% of the patients and 70% of samples with successful DNA extraction. If several technical issues remain concerning sequencing quality, which mainly depends on the sampling quality after extraction/amplification, further improvement in the number of isolated cells or of the isolation process may improve the results. In this setting, FACS technology using a CM-specific marker may be suitable for CM isolation.^11^

One of the advantages of this technique is the feasibility of DNA extraction from these few cells using single-cell DNA extraction technology and next-generation sequencing of this generated CM-DNA. We succeeded to sequence DNA extracted from CMs and found a good correlation with blood variants.^8^ However, DNA concentration is a crucial point to ensure the success of sequencing. In samples with lower concentration (see Supplemental Table 3), more artefactual variants were detected, needing additional Sanger sequencing control. The lower concentration in RUN 2-3 samples could be explained by an additional freezing-defreezing cycle, a lower number of cardiomyocytes per sample, and also by a longer conservation duration after which sampling had been processed. Thus, processing the cardiomyocytes samples quickly after sampling may improve the sequencing quality and robustness of the technique.

The parallel interpretation of variants from blood DNA and CM-DNA would allow to detect additional variants in CMs. After a mandatory step of validation by Sanger sequencing, this finding may support the hypothesis of mosaism explaining the disease in patients with negative genetic testing in usual conditions (blood DNA). In cardiomyopathies, this mechanism is poorly described. Few cases reports presented nonheterozygous variants associated with cardiomyopathy.^12^^,^^13^ In all the cases presented, the children were heterozygous, and 1 parent carried the variant at a mosaicism state, but only blood samples have been analyzed. Because somatic heart mosaicism is currently not investigated, this new technique could be used to explore this hypothesis.

However, some variants could have been generated during the whole genome amplification and sequencing phases (especially in presence of low DNA concentration), leading to potential false-positive variants, together with difficulties in reading alignment caused by amplification adapters because of non-expected sequences. Multiple tissues sampling and sequencing, especially in tissues from mesoderma as muscle or skin, could also argue the presence of a mosaism in cardiac tissue. In 1 of our patients, only 1 sample presented with a variant not found in leukocytes,^8^ but the buccal cells collected in a second time did not allow to conclude because the quality of Sanger electropherogram was too low to be interpretable. Thus, it was difficult to conclude for this case. In future procedures, more independent samples should be collected.

The absence of detection of additional variants in CMs in mort patients could be explain by some technical limitations, (1) some regions were not covered in NGS sequencing of samples comparing to usual blood sequencing, (2) we could not exclude variants in regions nonsequenced (deep intron or other genes), (3) the copy-number variants were not detected by this method in CMs, (4) we did not performed Sanger sequencing confirmation for all the additional variants found in samples (just on variants with sufficient quality).

### Limitations

The main issue remains the risk of residual contamination by leukocytes, which were present in many samples when looked for. Leukocyte contamination, even if reduced by the repeated filtering, may represent an issue for genetic analysis on a DNA not fully extracted from CMs. In that setting, more specific methods of CM isolation, such as FACS,^11^ may be particularly interesting to achieve more specific isolation, even if challenging because of the large size of adult human cardiomyoytes (700-1500 mm^3^).^14^ Cell isolation by sorting cell nucleus using sedimentation, immunolabeling with antibodies against pericentriolar material 1, and subsequent flow cytometry should also be tested in the future.^15^

Second, when only 1 cell is sampled, the amplification step may generate artefactual variants, and a unique cell might not be representative for the whole tissue of interest, but this concerned only 1 case. Endomyocardial biopsy could allow an analysis at the organ level and be more sensitive to detect mosaicism.

Third, this technique will not allow cell cultures at the moment because extraction and isolation of CMs led to major cellular damages; therefore, cells are no more unable to adhere. The same limitations due to cell structure damage will also concern immunohistochemistry or functional studies, such as patch-clamp techniques, for example. Transcriptional studies may also be limited by the risk of early transcriptional changes due to cell damage during CM isolation. However, the whole process of cell isolation could be achieved in less than 15 minutes by an experienced operator. The development of single-cell RNA sequencing technologies may enable the use of this technique for transcriptomic analysis in the near future.

### Conclusion

In conclusion, we present a novel mini-invasive technique of sampling of human living CMs allowing genetic analysis. The collected cells did not allow cell culture for now, but this technique open new sources of CMs for research and potential mosaism detection.

## Data Availability

Data are available on request at mauryjphil@hotmail.com.

## ORCID

Philippe Maury: https://orcid.org/0000-0002-4800-1841

## Conflict of Interest

The authors declare no conflicts of interest.
