# Supplementary material for: Methodology of DNA extraction and sequencing from living cardiomyocytes collected by catheter in humans
Source: Genet Med Open. 2025 Nov 18;4:103473. doi: 10.1016/j.gimo.2025.103473 (PMC12800430; doi:10.1016/j.gimo.2025.103473)
Supplement: Supplemental Material [file mmc1.docx]

**Supplementary Table 1: List of genes sequenced (coding regions +/-10 bp) :**

| Gene name | chr | NM | HGNC ID |
| --- | --- | --- | --- |
| *ABCC9* | chr12 | NM_005691.5 | HGNC:60 |
| *AKAP9* | chr7 | NM_005751.4 | HGNC:379 |
| *ANK2* | chr4 | NM_001148.4 | HGNC:493 |
| *CACNA1C* | chr12 | NM_199460.3 | HGNC:1390 |
| *CACNA1B* | chr9 | NM_000718.3 | HGNC:1389 |
| *CACNA2D1* | chr7 | NM_000722.3 | HGNC:1399 |
| *CACNB2* | chr10 | NM_201596.2 | HGNC:1402 |
| *CALM1* | chr14 | NM_006888.4 | HGNC:1442 |
| *CALM2* | chr2 | NM_1305624.1 | HGNC:1445 |
| *CASQ2* | chr1 | NM_001232.3 | HGNC:1513 |
| *CAV3* | chr3 | NM_033337.2 | HGNC:1529 |
| *CTNNA3* | chr10 | NM_013266.3 | HGNC:2511 |
| *DSC2* | chr18 | NM_024422.3 | HGNC:3036 |
| *DSG2* | chr18 | NM_001943.3 | HGNC:3049 |
| *DSP* | chr6 | NM_004415.3 | HGNC:3052 |
| *DES* | chr2 | NM_001927.3 | HGNC:2770 |
| *DPP6* | chr7 | NM_130797.4 | HGNC:3010 |
| *GJA1* | chr6 | NM 000165.4 | HGNC:4274 |
| *GJA5* | chr1 | NM_005266.6 | HGNC:4279 |
| *GPD1L* | chr3 | NM_015141.3 | HGNC:28956 |
| *HCN4* | chr15 | NM_005477.2 | HGNC:16882 |
| *JUP* | chr17 | NM_002230.2 | HGNC:6207 |
| *KCNA5* | chr12 | NM_002234.3 | HGNC:6224 |
| *KCNE1* | chr21 | NM_000219.5 | HGNC:6240 |
| *KCNE2* | chr21 | NM_172201.1 | HGNC:6242 |
| *KCNJ2* | chr17 | NM_000891.2 | HGNC:6263 |
| *KCNH2* | chr7 | NM_000238.3 | HGNC:6251 |
| *KCNQ1* | chr11 | NM_000219.3 | HGNC:6294 |
| *KCND3* | chr1 | NM_004980.4 | HGNC:6239 |
| *KCNE3* | chr11 | NM_005472.4 | HGNC:6243 |
| *KCNE5* | chrX | NM_012282.2 | HGNC:6241 |
| *KCNJ5* | chr11 | NM_000890.3 | HGNC:6266 |
| *KCNJ8* | chr12 | NM_004982.2 | HGNC:6269 |
| *LMNA* | chr1 | NM_170707.2 | HGNC:6636 |
| *NPPA* | chr1 | NM_006172.3 | HGNC:7939 |
| *NKX2-5* | chr5 | NM_004387.3 | HGNC:2488 |
| *NOS1AP* | chr1 | NM_14697.2 | HGNC:16859 |
| *PKP2* | chr12 | NM_004572.3 | HGNC:9024 |
| *PLN* | chr6 | NM_002667.4 | HGNC:9080 |
| *PRKAG2* | chr7 | NM_016203.3 | HGNC:9386 |
| *RANGRF* | chr17 | NM_016492.4 | HGNC:17679 |
| *RYR2* | chr1 | NM_001035.2 | HGNC:10484 |
| *SCN5A* | chr3 | NM_198056.2 | HGNC:10593 |
| *SCN10A* | chr3 | NM_ 006514.3 | HGNC:10582 |
| *SCN1B* | chr19 | NM_001037.5 | HGNC:10586 |
| *SCN2B* | chr11 | NM_004588.4 | HGNC:10589 |
| *SCN3B* | chr11 | NM_018400.3 | HGNC:20665 |
| *SCN4B* | chr11 | NM_174934.3 | HGNC:10592 |
| *SLMAP* | chr3 | NM_1304420.2 | HGNC:16643 |
| *SNTA1* | chr20 | NM_003098.2 | HGNC:11167 |
| *TGFB3* | chr14 | NM_003239.2 | HGNC:11769 |
| *TMEM43* | chr3 | NM_24334.2 | HGNC:28472 |
| *TRDN* | chr6 | NM_6073.3 | HGNC:12261 |
| *TRPM4* | chr19 | NM_17636.3 | HGNC:17993 |
| *TRPM7* | chr15 | NM_17672.5 | HGNC:17994 |

**Supplementary Table 2: Summary of the patients and samples**

| Patient Inclusion number | Statut dans Flow Chart | Total number of samples (tubes) available per patient | Extraction failure (number of samples) | Number of tubes sequenced | Number of samples/tube sequenced included in the analysis | Remaining samples/tubes not used |
| --- | --- | --- | --- | --- | --- | --- |
| 1 | Sampling failure | 0 |  |  | 0 |  |
| 2 | Sampling failure | 0 |  |  | 0 |  |
| 3 | Sampling failure | 0 |  |  | 0 |  |
| 4 | Sampling failure | 0 |  |  | 0 |  |
| 5 | Sampling diluationerror | 0 |  |  | 0 |  |
| 6 | Sampling diluationerror | 0 |  |  | 0 |  |
| 7 | Sampling diluationerror | 0 |  |  | 0 |  |
| 8 | Sampling diluationerror | 0 |  |  | 0 |  |
| 9 | Sampling diluationerror | 0 |  |  | 0 |  |
| 10 | Sampling diluationerror | 0 |  |  | 0 |  |
| 11 | Sampling diluationerror | 0 |  |  | 0 |  |
| 12 | Sampling diluationerror | 0 |  |  | 0 |  |
| 13 | Sampling diluationerror | 0 |  |  | 0 |  |
| 14 | Sampling diluationerror | 0 |  |  | 0 |  |
| 15 | Sampling diluationerror | 0 |  |  | 0 |  |
| 16 | Sampling diluationerror | 0 |  |  | 0 |  |
| 17 | Sampling diluationerror | 0 |  |  | 0 |  |
| 18 | Included | 3 | 0 | 2 | 2 | 1 |
| 19 | Included | 1 | 0 | 1 | 1 | 0 |
| 20 | Sequencingfailure | 1 | 0 | Sequencingfailure (1) | 0 | 0 |
| 21 | Included | 28 | 21 | 1 | 1 | 6 |
| 22 | Included | 17 | 7 | 7 | 7 | 3 |
| 23 | Included | 8 | 4 | 4 | 4 | 0 |
| 24 | Included | 9 | 2 | 7 | 7 | 0 |
| 25 | Included | 9 | 3 | 6 | 6 | 0 |
| 26 | Included | 8 | 3 | 5 | 5 | 0 |
| 27 | Included | 6 | 3 | 3 | 3 | 0 |
| 28 | Included | 7 | 3 | 3 | 3 | 1 |
| 29 | Excluded for comparison (blood missing) | 9 | 0 | Not included for CM/blood comparison | 0 | 8 |
| 30 | Included | 5 | 1 | 4 | 4 | 0 |
| 31 | Sequencingfailure | 5 | 4 | Sequencingfailure (1) | 0 | 0 |
| 32 | Excluded for comparison (blood missing) | 4 | 0 | Not included for CM/blood comparison | 0 | 3 |
| 33 | Excluded for comparison (blood missing) | 10 | 5 | Not included for CM/blood comparison | 0 | 0 |
| 34 | Extraction failure | 1 | 1 | 0 | 0 | 0 |
| 35 | Extraction failure | 1 | 1 | 0 | 0 | 0 |
| 36 | Extraction failure | 1 | 1 | 0 | 0 | 0 |
| 37 | Extraction failure | 1 | 1 | 0 | 0 | 0 |
| 38 | Extraction failure | 1 | 1 | 0 | 0 | 0 |
| 39 | Extraction failure | 1 | 1 | 0 | 0 | 0 |

**Methods of DNA extraction**

Cardiomyocytes have been treated in 2 set, in a first 23 cardiomyocytes samples (extraction and sequencing, RUN 1 in 2020) and a second set of 48 cardiomyocytes (extraction and 2 sequencing runs (RUN 2 and 3) in 2023).

A mean concentration of 2.02 ng/uL (SD 1.3) corresponding to a mean of 60,8 ng (SD39) of DNA per sample for run 2 and 3 and 3390+/-420 ng par sample for cardiomyoctes from run 1. The first set of extraction (RUN1) led to DNA concentration comparable with blood extracted DNA concentration. Extraction from run 2 and 3 led to lower concentration. This could be explained by an additional freezing-defreezing cycle in run2_3 and by the selection of the best sample (more cardiomyocytes “a priori” by sample) in the RUN 1.

**Sequencing performances**

This has led to a lower sequencing quality in RUN 2+3, comparing with RUN 1. Regarding the sequencing performance, mean coverage was lower in run 1 comparing to run 2 and 3 and blood run. Cardiomyocytes runs presented 10 -11, 5% of not covered region, defined as a coverage <30x (Table 2).

**Supplementary Table 3: quality of sequencing runs.**

|  | **RUN 1** | **RUN 2 ET 3** | **BLOOD RUN** |
| --- | --- | --- | --- |
| Read number per sample | 1448678±149788 | 2145520 +/- 599708 | 1927391±155509 |
| Duplicates | 5% | 10.5% (+/-3.9%). | <1% |
| Inside targets | 88±2 % | 72.6% +/-2.9% | 95±0.5% |
| Number of region with a coverage (>30x) | 10%  91+/-50 | 11,5% (117/1019)+/-15%. | 0 |
| Mean DNA concentration (ng/uL) | 113+/-14 | 2.02 +/-1,3 | 120+/-38 |
| Mean coverage | 263±191 x | 617.27 ± 174.04 x. | 623±77x |

We have observed an enrichment of variant with VAF >95% in the runs 2+3 comparing to the cardiomyocytes sequenced in run 1. For example, in samples from patient 18, VAF>95% represented 37% and 38% of respectively blood and Cardiocyte run 1 sequencing comparing to 77% of Run2+3 sequencing. Thus, for Sanger Sequencing performed in order to confirm the presence of a variant in Cardiocyte vs blood, we didn’t consider VAF>95% variants. We performed first confirmation after result from run 1 (Table 3), and then taking in consideration this first results, we decided to confirmed in run 2 and 3 only variants with a VAF 45- 55%, and not the insertion deletion in repeated regions.

**Supplementary Table 4: Variants identification and Sanger confirmation.**

| NGS sequencing batch | Patient-Sample | Additional variant: gene | HGNC ID | Additional variant: genomic nomenclature GRCh37 (hg19) | Additional variant: HGVS DNA on transcript | ACMG Classification (Intervar) | VAF | Death | Sanger confirmation |
| --- | --- | --- | --- | --- | --- | --- | --- | --- | --- |
| **1** | **20-1** | ***DPP6*** | HGNC:3010 | NC_000007.13:g.154677425T>Cchr7:g.154677425T>C | NM_130797.4:c.2216T>C | **VUS:**  **PM2**  **PP3_Moderate** | **51%** | **37x** | **Yes** |
| 1 | 23-1 | *RYR2* | HGNC:10484 | NC_000001.10:g.237754253_237754254insCCAAACACACCCAACAC chr1:g.237754253_237754254insCCAAACACACCCAACAC | [NM_001035.3](https://www.ncbi.nlm.nih.gov/nuccore/NM_001035.3):c.4121_4122insCCAAACACACCCAACAC | Pathogenic : PVS1 PM2 | 36% | 135x | No |
| 1 | 26-1 | *DSP* | HGNC:3052 | NC_000006.11:g.7580619C>G  chr6:g.7580619C>G | NM_004415.3:c.4196C>G | VUS:PM2  BP4_Moderate | 42% | 38x | No |
| 1 | 20-1 | *DSC2* | HGNC:3036 | NC_000018.9:g.28669472T>A  chr18:g.28669472T>A | [NM_024422.6](https://www.ncbi.nlm.nih.gov/nuccore/NM_024422.6):c.560A>T | VUS: PM1 PM2 | 65% | 60x | No |
| 1 | 20-1 | *TRPM4* | HGNC:17993 | NC_000019.9:g.49674644T>C chr19:g.49674644T>C | [NM_017636.4](https://www.ncbi.nlm.nih.gov/nuccore/NM_017636.4):c.827T>C | VUS: PM1 | 48% | 42x | No |
| 1 | 20-1 | *TRPM4* | HGNC:17993 | NC_000019.9:g.49714049_49714050insCGGAGTCGCTCTCCCGCTTGTCCCTAGCGCGTGCC  chr19:g.49714049_49714050insCGGAGTCGCTCTCCCGCTTGTCCCTAGCGCGTGCC | [NM_017636.4](https://www.ncbi.nlm.nih.gov/nuccore/NM_017636.4):c.3411_3412insCGGAGTCGCTCTCCCGCTTGTCCCTAGCGCGTGCC | VUS: PM2 | 40% | 30x | No |
| 1 | 19-1 | *CACNA1C* | HGNC:1390 | NC_000012.11:g.2224475_2224476insAAACACACCCAACA  chr12:g.2224475_2224476insAAACACACCCAACA | [NM_000719.7](https://www.ncbi.nlm.nih.gov/nuccore/NM_000719.7):c.135_136insAAACACACCCAACA | Pathogenic :PVS1 PM2 | 51% | 37x | No |
| 2+3 | 24-1-D4 | *RYR2* | HGNC:10484 | NC_000001.10:g.237777669C>T  chr1:g.237777669C>T | [NM_001035.3](https://www.ncbi.nlm.nih.gov/nuccore/NM_001035.3):c.5241C>T | Likely benin: PM2 BP4 BP7 | 54% | 393x | No |
| 2+3 | 21-1- H8 | *DSP* | HGNC:3052 | NC_000006.11:g.7579739G>A  chr6:g.7579739G>A | [NM_004415.3](https://www.ncbi.nlm.nih.gov/nuccore/NM_004415.4):c.3316G>A | VUS: PM1 PM2 | 52% | 172x | No |
| 2+3 | 21-1- H8 | *DSP* | HGNC:3052 | NC_000006.11:g.7583538G>A  chr6:g.7583538G>A | NM_004415.3:c.6043G>A | VUS: PM1 PM2 | 53% | 239x | No |
| 2+3 | 21-2- H3- | *DSG2* | HGNC:3049 | NC_000018.9:g.29118718C>A chr18:g.29118718C>A | [NM_001943.5](https://www.ncbi.nlm.nih.gov/nuccore/NM_001943.5):c.1656C>A | Likely benin:  PM2 BP4 BP7 | 45% | 378x | No |
| 2+3 | 18-1- G1 | *PKP2* | HGNC:9024 | NC_000012.11:g.32949082_32949083insACACCCAACC  chr12:g.32949213_32949214insCACACCCAAC | [NM_004572.3](https://www.ncbi.nlm.nih.gov/nuccore/NM_004572.3):c.2319_2320insTTGGGTGTGG | Pathogenic : PVS1 PM2 | 57% | 597x | No |
| 2+3 | 18-1- E1- | *CACNA2D1* | HGNC:1399 | NC_000007.13:g.81641555T>C chr7:g.81641555T>C | [NM_000722.4](https://www.ncbi.nlm.nih.gov/nuccore/NM_000722.4):c.1277A>G | VUS: PM1 PM2 BP1 | 31% | 529x | No |
| 2+3 | 18-1- G1 | *CACNA2D1* | HGNC:1399 | NC_000007.13:g.81641555T>C chr7:g.81641555T>C | [NM_000722.4](https://www.ncbi.nlm.nih.gov/nuccore/NM_000722.4):c.1277A>G | VUS: PM1 PM2 BP1 | 75% | 236x | No |
| 2+3 | 22-1- C2- | *DSP* | HGNC:3052 | NC_000006.11:g.7584617C>T  chr6:g.7584617C>T | [NM_004415.3](https://www.ncbi.nlm.nih.gov/nuccore/NM_004415.3):c.7122C>T | Benin : BA1 BP4 BP6 BP7 BS1 | 69% | 504x | Yes (already known in blood) |
| 2+3 | 22-2-A4- | *DSP* | HGNC:3052 | NC_000006.11:g.7584617C>T chr6:g.7584617C>T | [NM_004415.3](https://www.ncbi.nlm.nih.gov/nuccore/NM_004415.3):c.7122C>T | Benin : BA1 BP4 BP6 BP7 BS1 | 78% | 265x | Yes (already known in blood) |
| 2+3 | 22-2- B4- | *DSP* | HGNC:3052 | NC_000006.11:g.7584617C>T chr6:g.7584617C>T | [NM_004415.3](https://www.ncbi.nlm.nih.gov/nuccore/NM_004415.3):c.7122C>T | Benin : BA1 BP4 BP6 BP7 BS1 | 63% | 442x | Yes (already known in blood) |

VUS: variant of uncertain significance

We also observed a variant allele frequency (VAF) variability (example table 4) in variants detected in both blood and different CM samples for the same patient especially regarding the sample sequenced in RUN 2+3.

**Supplementary Table 5: variant allele frequency for a same variant for patient 19**, in leukocytes (line 1) and in different CM-samples (4 last lines).

| NGS sequencing batch |  |  |  |  | Coverage (x) | Alternative basecoverage (x) | VAF |
| --- | --- | --- | --- | --- | --- | --- | --- |
|  | Leuko-19 | *DSG2* | c.2137G>A | p.(Glu713Lys) | 562 | 250 | 44,00% |
| 1 | CM-19-2 | *DSG2* | c.2137G>A | p.(Glu713Lys) | 225 | 103 | 46,00% |
| 2+3 | CM-A6-19-2 | *DSG2* | c.2137G>A | p.(Glu713Lys) | 385 | 124 | 32,21% |
| 2+3 | CM_F9-19-1 | *DSG2* | c.2137G>A | p.(Glu713Lys) | 418 | 231 | 55,26% |
| 2+3 | CM_H1-19-2 | *DSG2* | c.2137G>A | p.(Glu713Lys) | 432 | 296 | 68,52% |

In conclusion, DNA concentration is a crucial point to ensure the success of the sequencing. Indeed, in run 2 and 3 due to low DNA concentration we observed a higher number of probably artefactual variants linked with a higher level of duplicated sequences. Additionally, we also observed a variant allele frequency (VAF) variability between heterozygous variants detected in all samples (example in Table 3). Thus, it’s challenging to define a quality threshold of a VAF and coverage before confirming these variants by sanger sequencing, to avoid a large among of Sanger confirmations. For run 2 and 3, which presented a lower quality, we only confirmed missense variants with a VAF between 45 and 55% and a coverage over 100x for Sanger sequencing confirmation.
